# Decreased IL-7 Responsiveness Is Related to Oxidative Stress in HIV Disease

**DOI:** 10.1371/journal.pone.0058764

**Published:** 2013-03-07

**Authors:** Magdalina Kalinowska, Douglas A. Bazdar, Michael M. Lederman, Nicholas Funderburg, Scott F. Sieg

**Affiliations:** 1 Albert Einstein College of Medicine, Bronx, New York, United States of America; 2 Case Western Reserve University and University Hospitals of Cleveland, Cleveland, Ohio, United States of America; University Hospital Zurich, Switzerland

## Abstract

HIV disease results in decreased IL-7 receptor expression and IL-7 responsiveness in T cells. To explore mechanisms of these deficiencies, we compared CD127 expression and IL-7 induction of P-STAT5 in T cells from HIV-infected persons with serum concentrations of cytokines (IL-7, IL-6 and IL-15), markers of microbial translocation (sCD14 and LPS), and with an indicator of oxidative stress (malondialdehyde (MDA) adducts). CD127 expression was directly related to IL-7 responsiveness in most CD8+ T cell subsets but not in CD4+ T cells from HIV-infected persons. MDA adducts were increased in serum of HIV-infected patients and were inversely related to IL-7 responsiveness in CD8+ T cells and in central memory CD4+ T cells. Incubation of T cells from healthy controls with hydrogen peroxide resulted in impairments in IL-7 induction of P-STAT5. These findings suggest that oxidative stress that is characteristic of HIV disease could contribute to impairments in IL-7 responsiveness and disrupt T cell homeostasis.

## Introduction

IL-7 is an important cytokine for immune system homeostasis. IL-7 promotes T cell survival and mediates homeostatic proliferation in lymphopenic conditions [1], [2], [3]. The IL-7/IL-7 receptor axis is perturbed in HIV disease such that serum levels of IL-7 cytokine are increased [4] while T cell expression of IL-7 receptor is diminished [5], [6], [7], [8], [9], [10]. T cells from HIV-infected persons also tend to display poor intracellular signaling responses to IL-7 stimulation, particularly as measured by phosphorylation of STAT-5 [5], [11], [12], [13]. These perturbations in IL-7 responsiveness could contribute to HIV pathogenesis and adversely affect T cell reconstitution during administration of anti-retroviral therapy. Notably, both IL-7-induced P-STAT5 signaling and CD127 receptor expression have been linked to T cell recovery during anti-retroviral therapy administration [14], [15], [16], [17].

The molecular mechanisms that underlie impaired IL-7 responsiveness at the single-cell level are not fully discerned. It is plausible that diminished IL-7 receptor expression (CD127) is a key determinant of reduced IL-7 responsiveness in HIV disease. In support of this possibility, some studies have found a tight correlation between IL-7-induced P-STAT5 signaling and CD127 expression, particularly among CD8 cells [11]. Nonetheless, isolated CD8+CD127+ cells from HIV+ donors have defects in P-STAT5 signaling [13]. Moreover, the relationship between CD127 expression and CD4 T cell responses to IL-7 in cells from HIV-infected persons is less clear [18] and additional studies of these cells have implicated deficiencies that may occur downstream of the receptor [12]. Here, we assessed the relationships between CD4 and CD8 expression of CD127 and P-STAT5 induction by IL-7.

The physiologic effects of HIV infection that account for poor IL-7 responsiveness and for decreased CD127 expression *in vivo* are poorly defined. One hypothesis stems from the increased serum IL-7 levels that occur in HIV-infected persons, especially among persons with low CD4 cell counts [4]. Increased exposure to IL-7 *in vivo* could result in IL-7 receptor downmodulation, leading to reduced responsiveness to further stimulation with IL-7. Alternatively, immune activation has also been linked to decreased CD127 expression in T cells from HIV-infected persons [7]. In addition, we hypothesized that cytokine responsiveness could be reduced by oxidative stress that is elevated in the setting of HIV disease [19], [20]. The latter is plausible since oxidative stress can influence a variety of signaling pathways and in theory, could provide a mechanism of signaling regulation that is independent of CD127 receptor expression levels.

Malondialdehyde (MDA) adducts provide evidence of oxidative stress *in vivo* and increased detection of MDA adducts in plasma has been linked to aging and to HIV disease [19], [20], [21], [22], [23], [24]. MDA adducts are products of lipid peroxidation mediated by reactive oxygen species. Notably, these toxic aldehydes are highly reactive with protein, DNA and lipids and consequently, can mediate biological effects by modifying these targets. Examples of molecules that can be modified by MDA include low-density lipoprotein, elastin and collagen, which may have implications for cardiovascular disease [25], [26], [27]. Here, we find that MDA adducts are increased in plasma of HIV-infected persons and inversely correlated with IL-7 responsiveness. Our observations suggest that oxidative stress is a potential contributor to poor IL-7 responsiveness in HIV infection.

## Materials and Methods

### Cells

Peripheral blood samples were collected from HIV^+^ and HIV^-^ adult volunteers. All subjects signed written consent and all studies were approved by the University Hospitals of Cleveland internal review board. Whole blood was used to assess CD127 expression in T cell subsets and peripheral blood mononuclear cells (PBMC) were isolated for in vitro studies of IL-7 responsiveness. To obtain PBMC, blood was drawn in heparin-coated tubes and centrifuged over a ficoll cushion. PBMC were cultured in complete medium consisting of RPMI 1640 (BioWhittaker, Walkersville, MD) supplemented with 10% FBS (Sigma Aldrich, St. Louis, MO), 0.4% L-Glutamine (BioWhittaker), 0.4% Penicillin/Streptomycin (BioWhittaker), and 0.4% HEPES (BioWhittaker). In some studies, CD3+ T cells were purified from PBMC using a negative selection magnetic bead kit (Miltenyi) and AutoMACS technology. Purity was greater than 97% in each of 3 different donors as determined by flow cytometric analyses.

### Subjects

The subjects consisted of 19 viremic HIV+ donors (median plasma HIV RNA  = 17480 copies/ml; range  = 451–122990 copies/ml), 19 aviremic HIV+ donors (VL<50 copies/ml) and 15 healthy controls). Thirty-seven percent of the viremic donors and 100% of the aviremic donors were receiving anti-retroviral therapy at the time of the study. The median CD4 cell count for all HIV+ donors was 473 cells/µL, whereas the median CD4 cell counts for viremic and aviremic subjects were 288 and 629 cells/µL, respectively. The median age of the healthy controls, aviremic subjects and viremic subjects was 38.5, 41.9 and 43 years old, respectively; age differences between groups were not statistically significant.

### P-STAT5 and CD127 analyses

To measure P-STAT5 induction by IL-7, PBMC were incubated in 24 well plates at 1×10^6^ cells/well with or without rIL-7 (1 ng/ml; Cytheris) for 15 minutes. Anti-CD8 Pacific Blue (BD Pharmingen) was added to some cell cultures during the 15 minute incubation at 2 µg/ml. Cells were then fixed with 100 µl of 16% ultrapure grade formaldehyde (Polysciences Inc.) and incubated an additional 10 minutes. After incubation cells were washed with PBS, vortexed, and incubated with 500 µl of 90% methanol at −20°C for 30 minutes. Cells were washed twice, re-suspended in 50 µl of PBS/0.2% BSA buffer and stained with anti-CD3 PerCP (BD Bioscience), anti-CD45RA PE (BD Pharmingen), anti-CD27 FITC (BD Bioscience) and either mIgG1 Alexa Fluor 647 (BD Pharmingen) or anti-Stat5 (pY694) Alexa Fluor 647 (BD Phosflow) for 1 h on ice. In some experiments, anti-CD4 Pacific Blue, (BD Pharmingen) was included in the stain rather than anti-CD8. Cells were analyzed on a BD LSRII flow cytometer.

To assess CD127 expression, whole blood (200 µl/tube) was stained with anti-CD4 Pacific Blue (BD Pharmingen), anti-CD8 Alexa Fluor 700 (BD Pharmingen), anti-CD3 PerCP (BD Bioscience), anti-CD45RA APC (BD Pharmingen), anti-CD27 FITC (BD Bioscience) and either mIgG1 PE or anti-CD127 PE (BD Pharmingen). Blood was incubated with antibodies for 10 minutes at room temperature, treated with BD FACS lyse solution (BD Bioscience) and washed twice in PBS/1% BSA, 0.1% sodium azide prior to analyses on a BD LSRII flow cytometer.

### Hydrogen peroxide treatment and annexin V stain

PBMC from healthy donors were incubated in complete medium (2×10^6^ cells/ml). H_2_O_2_ (9.8 or 98 µM) was added for 30 minutes prior to addition of IL-7. Cells were then prepared as above for flow cytometric analysis of P-STAT5 expression and in some studies for annexin V staining to measure apoptosis.

For annexin V staining, cells were washed, re-suspended in annexin binding buffer and stained with annexin V reagent (annexin V-PE apoptosis detection kit; BD Bioscience) for 15 min in the dark. Cells were then analyzed on an LSRII flow cytometer within 1 h of staining. Cells incubated in medium alone, H_2_O_2_ alone or IL-7 alone served as comparisons.

### ELISAs

Serum was stored at −20°C until analyzed in batch by ELISA. Soluble CD14 in serum was measured by using the Quantikine sCD14 kit (R&D systems). LPS was measured with a limulus amebocyte lysate assay from Lonza (Basal, Switzerland) and cytokines (IL-6, IL-7 and IL-15 were measured with kits from R&D Systems. Serum MDA was measured with a commercially available kit from Cell Biolabs Inc., San Diego, CA.

### Statistical analysis

Kruskal-Wallis multigroup comparison and Mann Whitney tests were used to compare differences between HIV+ and HIV- donors. Exploratory correlation analyses were assessed by nonparametric Spearman's and by Partial correlation analyses (SPSS software).

## Results

### Decreased P-STAT5 signaling and CD127 expression in T cell subsets from HIV+ donors

**Figure 1:**
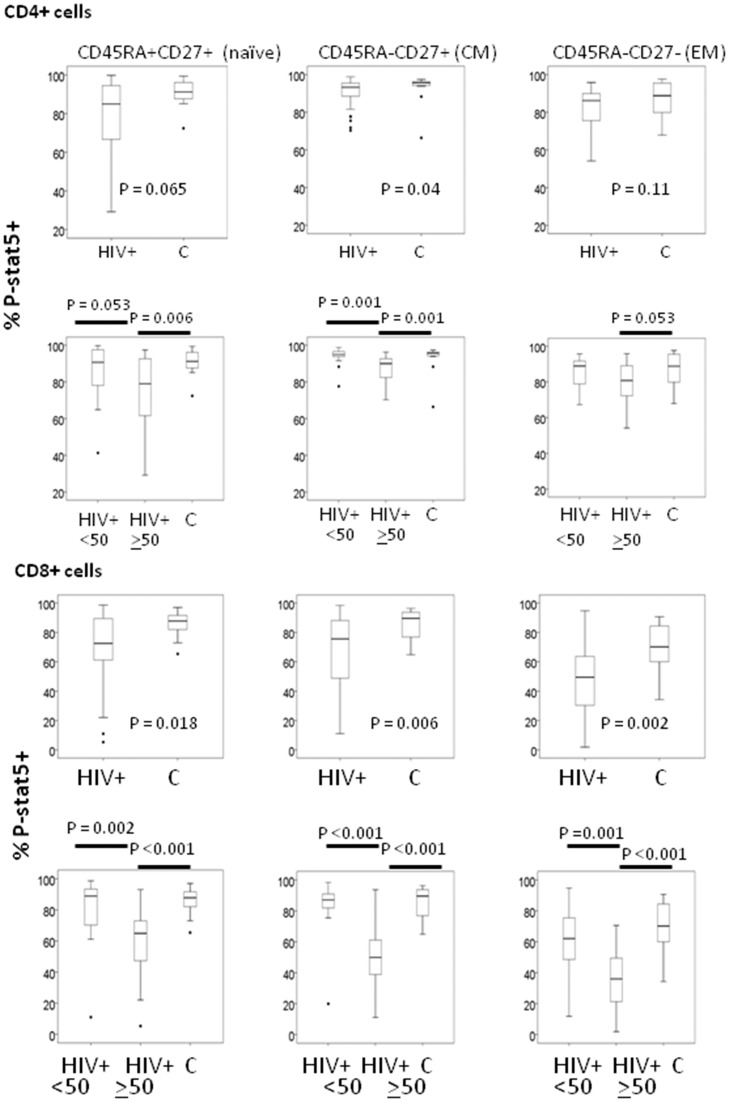
Diminished IL-7 induction of P-STAT5 in T cell subsets from HIV+ donors. PBMC were incubated for 15 min with rIL-7 (1 ng/ml) and cells were assessed by flow cyomtery for expression of P-STAT5. Responses of CD4 lymphocytes (A and B) and CD8 lymphocytes (C and D) are shown as box-and-whiskers plots indicating the median, the inter-quartile values (box), the range (whiskers) and the outliers (symbols). Data are represented for all HIV+ donors (A and C) or for viremic and aviremic donors (B and D). The percentage of P-STAT5 expression represents the percentages of P-STAT5+ cells that increased P-STAT5 expression above levels observed in unstimulated cells.

To assess IL-7 responsiveness in T cell subsets, PBMC were stimulated with rIL-7 and P-STAT5 was measured by intracellular flow cytometry. CD4+ and CD8+ T cell subsets were defined as enriched for naïve (CD45RA+CD27+), central memory (CM, CD45RA-CD27+) and effector memory (EM, CD45RA-CD27-) subsets. Terminal memory cells (CD45RA+CD27-) were not included in the analyses since these cells tend to express little IL-7 receptor, respond poorly to IL-7 stimulation and are relatively rare in the CD4+ T cell population. P-STAT5 signaling was diminished among T cells from HIV+ donors, particularly among viremic HIV+ donors (Figure 1 and Figure S1). Percentages of CD4 and CD8 cells that expressed CD127 also tended to be reduced in cells from HIV+ donors (not shown). Interestingly, among the CD127+ cells, we found evidence of diminished CD127 density in T cells from HIV+ donors (Figure S2). The diminished intensity of CD127 expression was observed in both CD4 and CD8+ T cells of viremic HIV+ donors and surprisingly, even in CD4+ cells from HIV+ aviremic donors.

### CD127 expression on CD8 cells, but not CD4 cells, is correlated with P-STAT5 signaling responses to IL-7 stimulation

**Figure 2:**
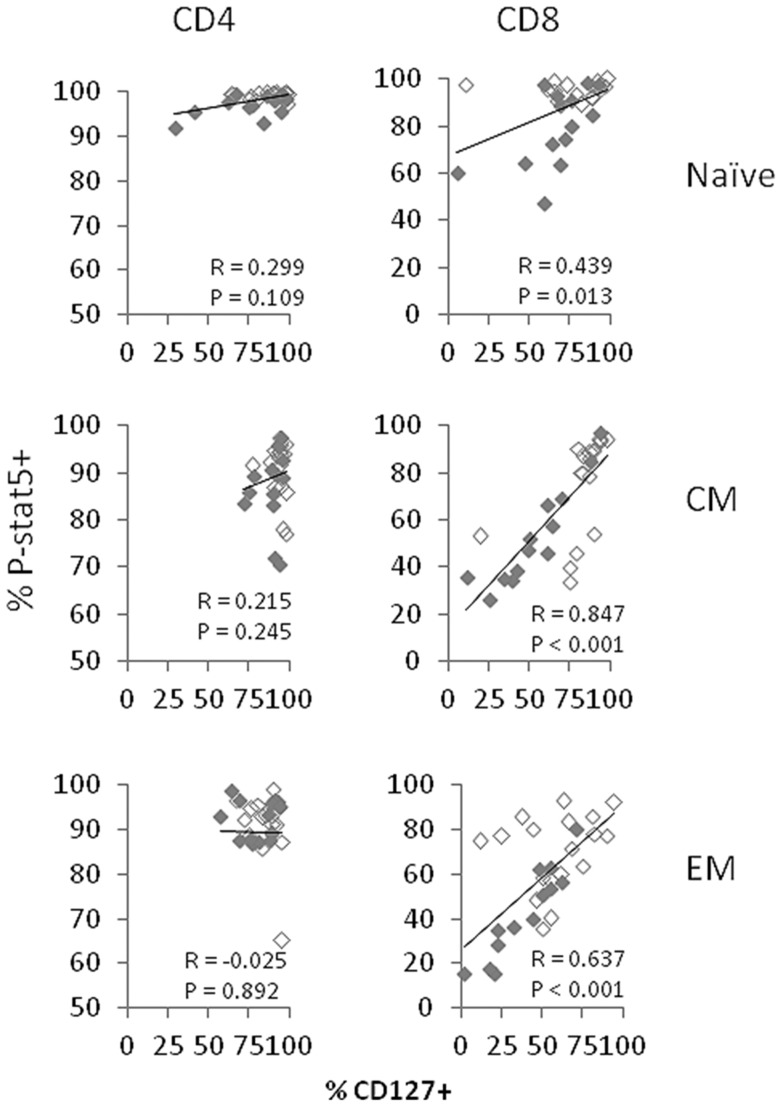
P-STAT5 induction is related to CD127 expression in CD8+ T cells. The relationship between IL-7-induced P-STAT+ cells and the percentage of CD127+ T cells in whole blood are shown for CD4 cells (left column) and CD8 cells (right column) within the indicated subsets. Open symbols represent aviremic subjects and closed symbols represent viremic subjects. Correlation coefficients and P values were determined by Spearman's correlations.

To assess the relationship between CD127 expression and P-STAT5 signal induction, we plotted the induction of P-STAT5 expression by IL-7 against the percentage of CD127+ cells or the CD127 MFI of CD127+ cells. Among all HIV+ donors, these indices tended to be directly correlated for CD8+ T cell subsets (Figure 2 and Table S1). These indices remained significantly correlated when the study subjects were separated into viremic and aviremic subjects and also tended to be directly related in healthy controls (not shown). In contrast, neither the percentages of CD127+ cells (Figure 2) nor the MFIs of CD127 expression (Table S1) were significantly related to IL-7 responsiveness in CD4+ T cell subsets, particularly in HIV+ donors. These relationships remained insignificant for viremic and aviremic subjects considered separately and also among viremic subjects who were not receiving anti-retroviral therapy (not shown). Notably, correlations in CM and EM CD8+ T cells for indices of P-STAT5 signaling and measures of CD127 expression remained significant in persons not receiving anti-retroviral therapy (r values >0.73 and p values <0.03). These data suggest that CD127 expression may be a less important predictor of IL-7 responsiveness in CD4 cells than in CD8 cells from HIV+ donors and that other mechanisms besides the levels of IL-7 receptor surface expression could play a role in diminished IL-7 responsiveness in HIV disease.

### Serum markers of microbial translocation and oxidative stress are increased in HIV-infected persons

**Figure 3:**
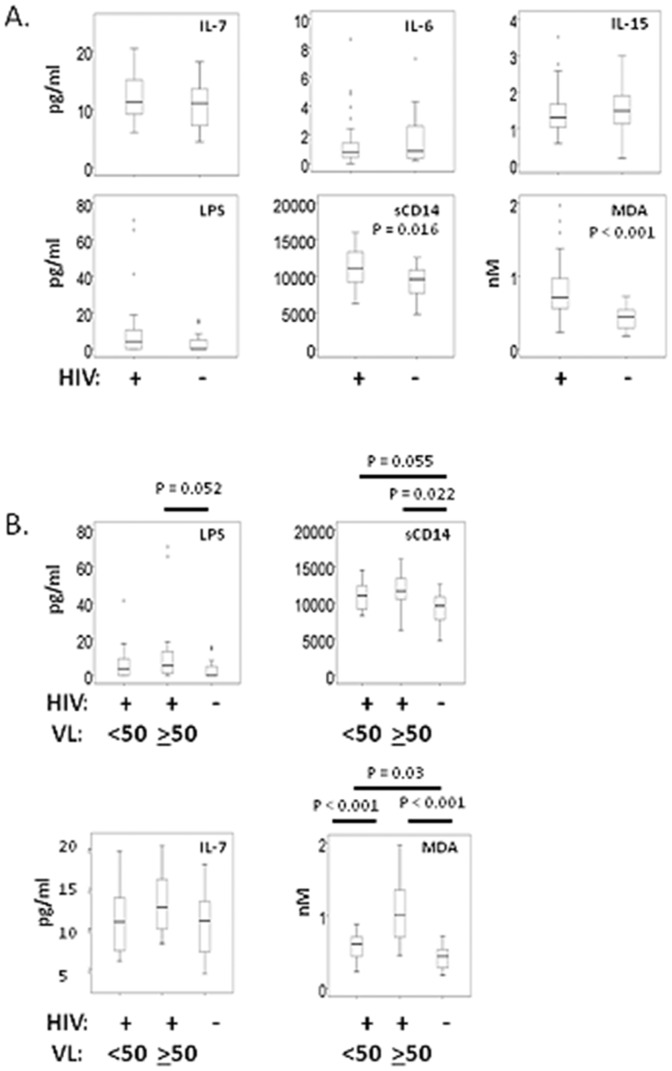
Increased sCD14 and MDA adducts in serum of HIV+ donors. Serum samples from HIV+ and HIV- donors were stored at −80°C until analyzed in batch with commercial ELISA kits. Data are shown comparing cytokine levels in all HIV+ donors to controls (A) or comparing viremic, aviremic and controls (B). Statistical significance was determined by Mann Whitney tests (A) or by Kruskal-Wallis Test for multi-group analysis and Mann Whitney (B).

To identify the mechanisms that could play a role in decreased IL-7 responsiveness in cells from HIV-infected persons, we assessed a number of serum markers. These included serum concentrations of IL-15 and IL-7 that may cause reduced CD127 expression [28], sCD14 and LPS, which are reflective of microbial translocation and immune activation [29], MDA adducts, which are products of lipid peroxidation due to oxidative stress [30], and IL-6, a marker associated with development of opportunistic infections and all-cause mortality in HIV disease [31]. Of these indices, sCD14 and MDA were significantly increased in serum from HIV+ donors compared to healthy controls (Figure 3). MDA was especially increased among viremic HIV+ donors, whereas sCD14 tended to be elevated in both viremic and aviremic subjects. IL-7 and LPS tended to be higher in viremic HIV+ donors than healthy controls but these differences were not statistically significant. Since previous studies implicated low CD4 cells as a predictor of IL-7 serum levels [4], we also compared HIV+ donors with CD4 counts below 350 to those above 350. Subjects with CD4 counts below 350 (n = 12) displayed statistically significant increases in serum IL-7 levels compared to healthy controls (p = 0.04). Neither IL-15 nor IL-6 serum levels were significantly different in our HIV+ donors compared to healthy controls. Overall, serum markers of microbial translocation and oxidative stress demonstrated the clearest differences between HIV+ and HIV- donors.

### Serum concentrations of MDA adducts are inversely related to P-STAT5 signaling and CD127 expression in T cells from HIV+ donors

**Figure 4:**
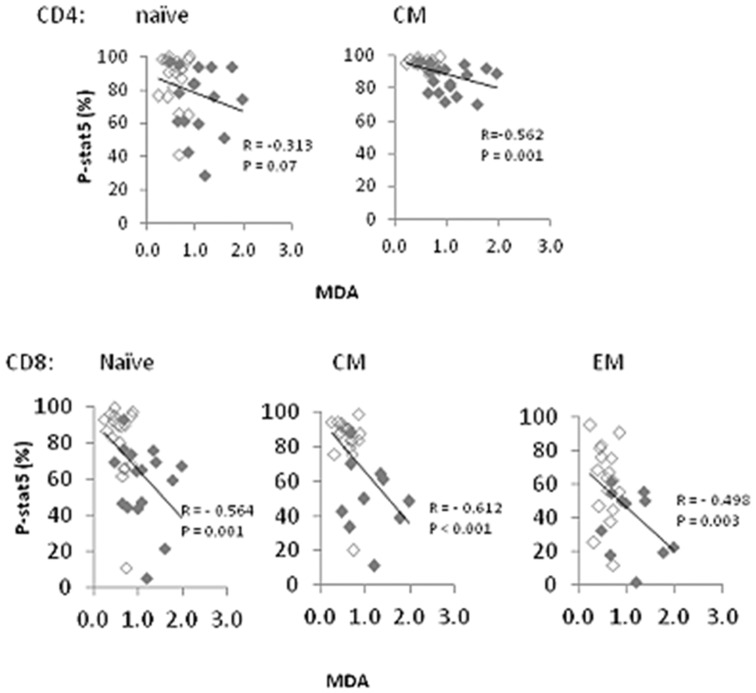
Serum concentrations of MDA adducts are inversely related to IL-7-induction of P-STAT5. Percent induction of P-STAT5 in the indicated T cell subsets was plotted against serum MDA adducts for each subject. Open symbols represent aviremic subjects and closed symbols represent viremic subjects. Correlation coefficients and P values were determined by Spearman's correlations.

The serum marker that was most consistently associated with measures of P-STAT5 signaling in cells from HIV+ donors was MDA adducts. MDA adducts were inversely related to P-STAT5 signaling in CM CD4+ T cells, with a similar trend that was not statistically significant observed in naïve CD4+ T cells (Figure 4). MDA adducts were also inversely related to P-STAT5 signaling in CD8+ T cells including naive, CM and EM subsets (Figure 4). Plasma HIV RNA also tended to be inversely related to induced P-STAT5 expression among cells from HIV+ donors (not shown) and also directly related to MDA adducts (r = 0.576, p<0.001). Therefore, we performed partial correlation analyses to control for the effects of viral load in the observed relationships between MDA and P-STAT5 signaling. Among CM CD4+ T cells, the relationship between MDA and P-STAT5 was of borderline significance (p = 0.050) when controlling for plasma HIV RNA copy numbers. Among CD8 cells, the relationships between MDA and P-STAT5 signaling remained significant for naïve, CM and EM subsets even after controlling for plasma HIV RNA copy numbers (not shown). Serum marker data were available for 8 subjects who were viremic and not receiving anti-retroviral therapy; no significant correlations were observed in this subset of patients between any serum markers and IL-7-induced P-STAT5 (not shown).

Serum MDA adducts also tended to correlate inversely with expression of CD127 on T cells (Table 1). Among CD4 cells, MDA adducts were inversely related to the percentage of CD127+ naïve cells (r = −0.568, p = 0.002) and the MFI of CD127 in EM T cells (r = -0.435, p = 0.023). Relationships of borderline significance were observed between MDA adducts and either CD127 MFI (p = 0.05) or percentage of CD127+ central memory T cells (p = 0.056). MDA adducts were also inversely related to the percentage of CD8+ naïve cells, EM cells and CD8+ CM cells (borderline significance) that expressed CD127 as well as the CD127 MFI of effector memory CD8 cells (Table 1). The relationships between percent CD127+ cells and MDA adducts remained significant for CD4+ naïve cells (p = 0.041) and for EM CD8+ T cells (p = 0.01) after controlling for plasma HIV RNA (partial correlation analyses) as did the relationship between CD127 MFI in CD8+ EM cells and MDA adducts (p = 0.019). Similar results were observed for the significant relationships reported above when using Partial correlations that controlled for IL-7, IL-15, LPS or CD4 cell count with the 2 exceptions that the relationship between MDA and the percentage of naïve CD8+ T cells that expressed CD127 was no longer significant when controlling for CD4 cell count (p = 0.067) or for LPS (p = 0.056). Overall, these data suggest that MDA adducts are related to CD127 expression and to IL-7 signaling deficiencies in T cells from HIV-infected persons in a manner that is independent of plasma HIV RNA copy numbers.

**Table 1:** Spearman correlations for relationships between MDA serum concentrations and measures of CD127 expression in T cell subsets.

Cells	Naïve (%CD127+)	Naïve (MFI CD127)	CM %CD127+	CM MFI CD127	EM %CD127+	EM MFI CD127
**CD4+**	R = −0.568 P = 0.002	R = −0.076 P = 0.711	R = −0.372 P = 0.056	R = −0.380 P = 0.05	R = −0.191 P = 0.331	R = −0.435 P = 0.023
**CD8+**	R = −0.453 P = 0.018	R = 0.157 P = 0.435	R = −0.380 P = 0.051	R = −0.343 P = 0.08	R = −0.517 P = 0.006	R = −0.546 P = 0.003

Among the other serum markers examined, only LPS was related to P-STAT5 signaling, and this relationship was restricted to CD8+ EM (r = 0.372, p = 0.028) and CD8+ CM cells (r = 0.341, p = 0.045); only the relationship within the CD8+ EM cells remained significant after controlling for plasma HIV RNA copy numbers (p = 0.037). Among CD8+ T cells, LPS was inversely related to the percentage of CM that expressed CD127 (r = −0.523, p = 0.004) and to MFI of CD127 on CM (r = −0.523, p = 0.004) and EM cells (r = −0.502, p = 0.006). These data suggest the possibility that microbial translocation could influence CD8 T cell responsiveness to IL-7, although it is notable that another marker thought to reflect microbial translocation, sCD14, was not significantly related to any of these indices.

Analyses of relationships between serum concentrations of IL-7 or IL-15 and IL-7 induced P-STAT5 signaling in T cells did not demonstrate any significant correlations. In general, serum IL-7 was not a good correlate of CD127 receptor expression with the exception of an inverse relationship with the percentage, but not MFI, of CD127+ CM CD4+ T cells (r = -0.543, p = 0.003 for percent CD127). Serum IL-7 was also not related to MDA among HIV+ donors (r = 0.04, p = 0.8). Serum IL-15 was inversely related to percentages of CD127+ naïve (r = −0.507, p = 0.008) and CD127+ CM CD4+ T cells (r = −0.409, p = 0.034) and to the percentage of CD127+ EM CD8+ T cells (r = −0.463, p = 0.015). It seems unlikely, however, that serum IL-15 could play an important role in IL-7 receptor deficiencies in HIV disease since it was not markedly increased in serum of HIV+ donors, even among those persons with viremia or among persons with CD4 cell counts <350 (not shown). These observations suggest that deficiencies in IL-7 mediated P-STAT5 signaling and in IL-7 receptor expression are not readily explained by increased exposure to IL-7 or IL-15 *in vivo*, at least as reflected by serum concentrations of these cytokines.

Since MDA was the most consistent predictor of IL-7 receptor signaling and surface expression, we asked what other indices were related to MDA serum levels. Both IL-6 and IL-15 serum levels were directly related to MDA in samples from HIV+ donors (Figure S3). Furthermore, MDA was directly related to plasma HIV RNA and inversely correlated with CD4 cell count (Figure S3). These data raise the possibility that oxidative stress might affect the expression of cytokines *in vivo* and are also supportive of a role for oxidative stress in HIV pathogenesis.

### Oxidative stress modifies IL-7 responsiveness in T cells

**Figure 5:**
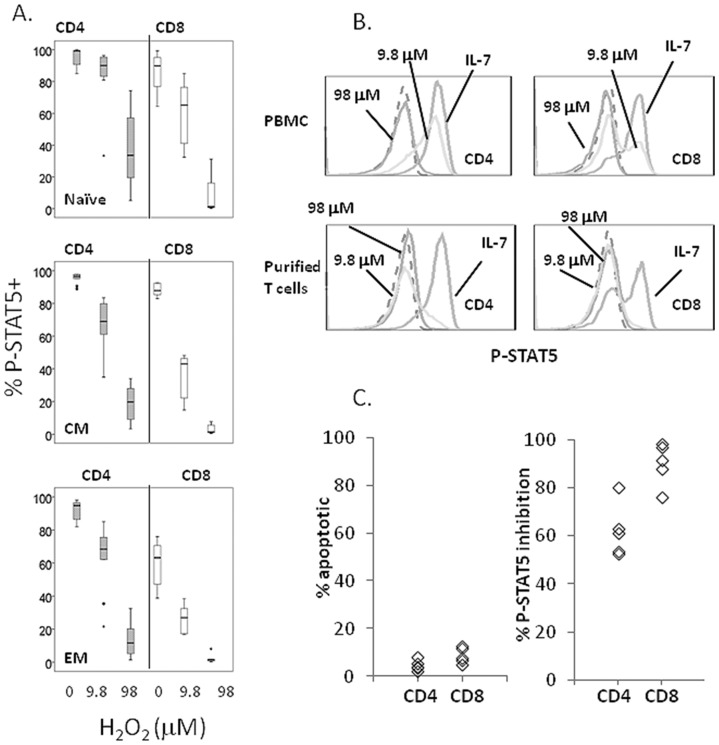
Hydrogen peroxide impairs IL-7 induction of P-STAT5 in T cell subsets. PBMC from healthy control donors were pre-incubated with H_2_O_2_ at the indicated concentrations for 30 minutes prior to stimulation with IL-7 (1 ng/ml). The sequential gating strategy included gating on live lymphocytes by forward and side scatter properties, removal of doublets (FSC-A vs FSC-H), gating on CD3+CD4+ cells or CD3+CD8+ cells and then gating on cells based on CD45RA and CD27 co-expression patterns to define subsets. Medians and inter-quartile ranges of P-STAT5+ cells within indicated subsets are represented by box-and-whisker plots for CD4+ T cells (n = 10) and CD8+ T cells (n = 6). Differences between H_2_O_2_-treated cells and cells incubated in medium alone were statistically significant at each concentration and for each cell type (p values <0.05). (A) P-STAT5 expression is shown in representative histograms of CD4+ (left column) and CD8+ (right column) T cells from either PBMC cell cultures (top) or purified T cells (>98% purity; bottom). Cells were incubated with H_2_O_2_ at the indicated molar concentrations for 30 min. or incubated in medium alone. Cells were then stimulated with IL-7 and assessed for intracellular P-STAT5 expression (1 ng/ml). Dashed histograms represent P-STAT5 expression in unstimulated cells. Data are representative of experiments from 3 different donors (B). PBMC from 5 different donors were used to assess the effects of H_2_O_2_ on T cell apoptosis and P-STAT5 induction. The percentages of total apoptotic CD4+ and CD8+ T cells were determined by annexin V staining during culture of PBMC with 98 µM H_2_O_2._ These percentages are compared to the percent inhibition of P-STAT5 signaling in PBMC that was observed after stimulation with IL-7 in the presence of 98 µM H_2_O_2_ (C). Note that the proportions of cells that show evidence of apoptosis are far lower than the proportions of cells with impaired P-STAT5 induction.

Of the serum markers studied, our observations suggest that oxidative stress is the best correlate of reduced IL-7 receptor expression and signaling in HIV disease. To investigate the possibility that oxidative stress could impair IL-7 signaling or receptor expression directly in T cells, PBMC from healthy controls were incubated *in vitro* with increasing concentrations of H_2_O_2_ and then stimulated with IL-7. Both CD4 and CD8 cells treated with H_2_O_2_ for 30 minutes developed poor responses to IL-7 stimulation as measured by P-STAT5 signaling (Figure 5A). The magnitude of P-STAT5 inhibition by H_2_O_2_ was more pronounced within the CD8 subset, suggesting that CD8 cells are more susceptible to the effects of oxidative stress than CD4 cells. Furthermore, naïve T cells appeared to be less readily affected by H_2_O_2_ than memory T cells within both the CD4 and CD8 subsets (Figure 5A). Notably, even when H_2_O_2_ was removed by 2 washes, deficiencies in IL-7 signaling persisted, although the inhibitory effect was reduced compared to cells maintained in hydrogen peroxide (not shown).

To determine if the effects of H_2_O_2_ were mediated directly on T cells or through an indirect mechanism, we compared IL-7 responses in PBMC and purified T cells pre-treated with H_2_O_2_. Pre-incubation of either PBMC or purified T cells with H_2_O_2_ resulted in impaired IL-7 induction of P-STAT5 (Figure 5B) among both CD4 and CD8+ T cells. Interestingly, the magnitude of the effect was more pronounced in the purified T cells at the lower H_2_O_2_ concentration, suggesting that the presence of other types of cells in the culture may actually provide some protective effects. These data clearly demonstrate that H_2_O_2_ can mediate direct effects on T cells.

To assess the possibility that H_2_O_2_ was toxic in these cell cultures and resulted in impairments in T cell function, we measured apoptosis with annexin V staining. Apoptosis was modestly increased by H_2_O_2_ in the time course of these experiments above the levels observed in cells incubated in medium alone (median percent apoptotic CD4+ T cells  = 2.6% and 4.0% for cells incubated in medium alone and with 98 µM of H_2_O_2,_ respectively, and the median percent apoptotic CD8+ T cells  = 4.7% and 7.5% for cells incubated in medium alone and with 98 µM H_2_O_2_, respectively). Moreover, the total percentages of apoptotic cells were considerably lower than the proportions of CD4 and CD8 cells that displayed impairments in P-STAT5 signaling after H_2_O_2_ incubation (Figure 5C). These data indicate that H_2_O_2_ toxicity is not a likely explanation for impaired IL-7 responses in T cells. Furthermore, we found no evidence that the concentrations of H_2_O_2_ that were used in these experiments had any effects on CD127 expression or any other measured markers on these T cells in 30 minute or even overnight incubations (not shown). These data raise the possibility that oxidative stress may be more than a surrogate marker of IL-7 signaling defects in HIV disease and could potentially cause direct inhibition of P-STAT5 signaling in T cells that occurs independent from cell surface CD127 expression.

## Discussion

It is well established that T cells from HIV-infected persons respond less readily to IL-7 stimulation than cells from healthy controls [5], [11], [12], [13], [30]. Our studies demonstrate a tight relationship between CD127 expression and IL-7 responsiveness among CD8+ T cells, especially central and effector memory cells. This relationship is also observed when considering the density of CD127 expression in memory CD8 cells. The reduced density of CD127 on CD8+ cells may help to explain previous observations indicating poor IL-7 responsiveness exclusively in CD8+CD127+ T cells from HIV+ donors compared to cells from healthy controls [13]. Furthermore, our studies suggest that oxidative stress may account in part, for reduced IL-7 responsiveness in CD8+ T cells from HIV-infected persons. This may be especially important among naïve CD8+ T cells, where the relationship between CD127 receptor expression and IL-7 responsiveness is less pronounced compared to memory cells.

The mechanism that explains reduced CD127 expression in CD8+ T cells from HIV-infected persons is still not entirely clear, but our results suggest that IL-7 serum levels are not a strong predictor of CD127 expression in these cells. In contrast, MDA adducts and LPS in serum tend to correlate inversely with CD127 expression in CD8+ T cells. Nonetheless, in our hands, neither exposure to LPS (not shown) nor exposure to H_2_O_2_ *in vitro* caused down-modulation of CD127 expression in T cells during overnight incubations (not shown) suggesting that these indices (MDA adducts and LPS) may be indirectly related to CD127 expression rather than causal. Overall, we favor a model whereby much of CD127 loss in the CD8+ T cell subset is the consequence of cellular maturation and differentiation of these cells caused by chronic immune activation. This raises the possibility that reduced IL-7 responsiveness in this subset, especially among the memory CD8+ T cells, could represent a switch in homeostatic cytokine requirements as a function of maturation and may not necessarily be reflective of a cellular defect. Notably, some degree of CD127 recovery on the surface of cells incubated *in vitro* in the absence of exogenous IL-7 is observed in T cells derived from either healthy donors or HIV-infected persons [5], [6], suggesting that some reversible mechanism of active down-modulation of CD127 surface expression appears to be operative *in vivo* and it is possible that this mechanism is influenced by HIV infection.

Deficiencies in CD4+ T cell responses to IL-7, especially among CM CD4+ T cells, may have important implications for HIV disease pathogenesis. Studies in SIV-infected monkeys suggest that CM CD4+ T cells have stem-cell like activity which contributes to reconstitution of effector memory cells in tissues. Disruption of CM T cell homeostasis is thought to underpin a loss in effector cells, leading to development of AIDS-defining illness [32]. Therefore, deficiencies in IL-7 responsiveness in the CM CD4+ T cell population could be especially important and contribute to homeostatic failure in HIV disease.

Our observations are consistent with previous studies that have demonstrated increased oxidative stress in HIV disease [19], [22], [33]. Notably, we found evidence of increased oxidative stress in both viremic and treated aviremic subjects compared to controls. The levels of MDA adducts; however, were significantly lower in persons with suppressed viremia compared to persons with incomplete control of viral replication, suggesting that control of viral replication reduces oxidative stress in HIV disease. MDA adducts also were directly related to IL-6 and IL-15 in serum of HIV-infected persons, raising the possibility that oxidative stress could influence the production of these cytokines. This concept is consistent with *in vitro* findings that oxidative stress can induce IL-6 production from various cell types [34], [35], [36], [37].

Importantly, our observations raise the possibility that increased oxidative stress could contribute to diminished responsiveness to IL-7 in T cells from HIV-infected patients as MDA adducts and IL-7 responsiveness are inversely related in these persons. T cells from healthy controls that are exposed to H_2_O_2_ *in vitro* rapidly develop defects in IL-7–induced signaling that are independent of CD127 expression. Coupled with previous observations demonstrating perturbations in glutathione in T cells from HIV-infected persons [38], [39], it seems likely that oxidative stress could contribute to deficiencies in IL-7 responsiveness in HIV disease. It should be noted, however, that the strength of the correlations between MDA and IL-7-induced P-STAT5 induction are only of borderline significance within the CM CD4+ cells when controlled for plasma HIV RNA levels and are absent in our small sample size of viremic HIV+ donors who were not receiving anti-retroviral therapy. Tighter correlations are noted within the CD8 subsets between MDA and IL-7-induced P-STAT5 and it is feasible that these relationships are due in part to the greater sensitivity of CD8 cells to the effects of oxidative stress as indicated by our in vitro experiments (Figure 5). Nonetheless, IL-7 responses are also tightly related to CD127 expression among these cells (Figure 2 and Table S1). Overall, it is likely that oxidative stress alone, will not fully explain all of the deficiencies in IL-7 responsiveness that occur in HIV disease. Further studies that discern the magnitude of oxidative stress in various T cell subsets and the capacity of various T cell subsets to cope with oxidative stress may provide additional insight into mechanisms that modify IL-7 responsiveness in HIV disease.

Reactive oxygen species (ROS) can cause damage to various molecules including DNA and proteins and also modify signaling activity in cells, including JAK/STAT activation. H_2_O_2_, for example, has been shown to inhibit JAK/STAT signaling induced by interferons in a hepatocellular carcinoma cell line cell by interfering with tyrosine kinase signaling [40] and nitric oxide also interferes with JAK/STAT activation [41], [42]. Previous studies suggest that oxidative stress can modify T cell activity including cytokine production and survival and may affect T cell subsets differently [43], [44], [45], [46]. Understanding the interplay between the IL-7/IL-7 receptor axis, oxidative stress and immune homeostasis may provide new insight into T cell pathogenesis and immune reconstitution in HIV disease.

## Supporting Information

Figure S1 **Percent increase in P-STAT5+ cells after IL-7 stimulation.** PBMC were incubated with rIL-7 for 15 min. and intracellular P-STAT5 expression was assessed by flow cytometry among CD45RA+CD27+ naïve cells, CD45RA-CD27+ central memory cells and CD45RA-CD27- effector memory cells for both the CD3+CD4+ (left columns) and CD3+CD8+ (right columns) subsets. A representative response from cells of a healthy control and HIV+ donor are shown. Two peaks were frequently observed in responding cells, especially among HIV+ subjects with subnormal responses to IL-7, providing rationale for evaluating percent positive cells as the measure of P-STAT5 induction.
(TIF)

Figure S2 **Reduced density of CD127 expression in T cells from HIV+ donors.** Freshly isolated whole blood was stained for expression of CD127 in T cell subsets. The mean fluorescence intensity was measured specifically on CD127+ cells that were identified with an isotype control background stain. Box-and-whiskers plots are shown for MFI of CD127 expression in CD4+ T cells (A and B) and CD8+ T cells (C and D). Data represent all HIV+ donors (A and C) or virmeic and aviremic donors (B and D).
(TIF)

Figure S3 **Serum MDA adducts are related to IL-6 and IL-15 cytokines and also correlated with clinical indices of disease progression.** Serum concentrations of IL-6 and IL-15, CD4 T cell counts and plasma HIV RNA were plotted against serum MDA adducts in HIV+ donors. Open symbols represent aviremic subjects and closed symbols represent viremic subjects. Correlation coefficients and P values were determined by Spearman's correlations.
(TIF)

Table S1 **Spearman's correlations indicating the relationships between CD127 MFI and P-STAT5 induction by IL-7 in T cell subsets from HIV+ donors.**
(TIF)
