# Supplementary figures and images for: Decreased IL-7 Responsiveness Is Related to Oxidative Stress in HIV Disease
Source: PLoS One. 2013 Mar 7;8(3):e58764. doi: 10.1371/journal.pone.0058764 (PMC3591367; doi:10.1371/journal.pone.0058764)

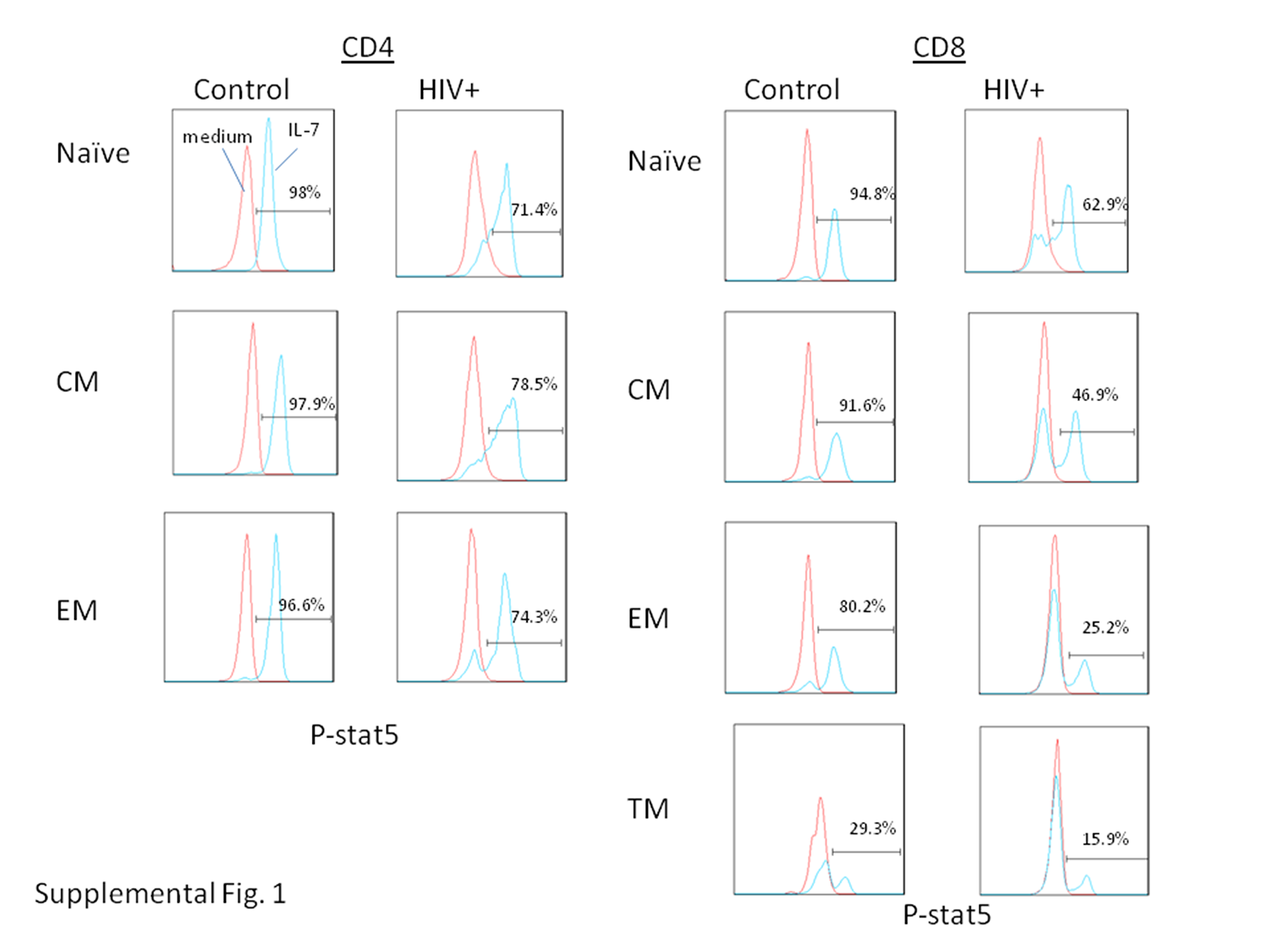

Supplement: Figure S1 — Percent increase in P-STAT5+ cells after IL-7 stimulation. PBMC were incubated with rIL-7 for 15 min. and intracellular P-STAT5 expression was assessed by flow cytometry among CD45RA+CD27+ naïve cells, CD45RA-CD27+ central memory cells and CD45RA-CD27- effector memory cells for both the CD3+CD4+ (left columns) and CD3+CD8+ (right columns) subsets. A representative response from cells of a healthy control and HIV+ donor are shown. Two peaks were frequently observed in responding cells, especially among HIV+ subjects with subnormal responses to IL-7, providing rationale for evaluating percent positive cells as the measure of P-STAT5 induction. (TIF) [file pone.0058764.s001.tif]

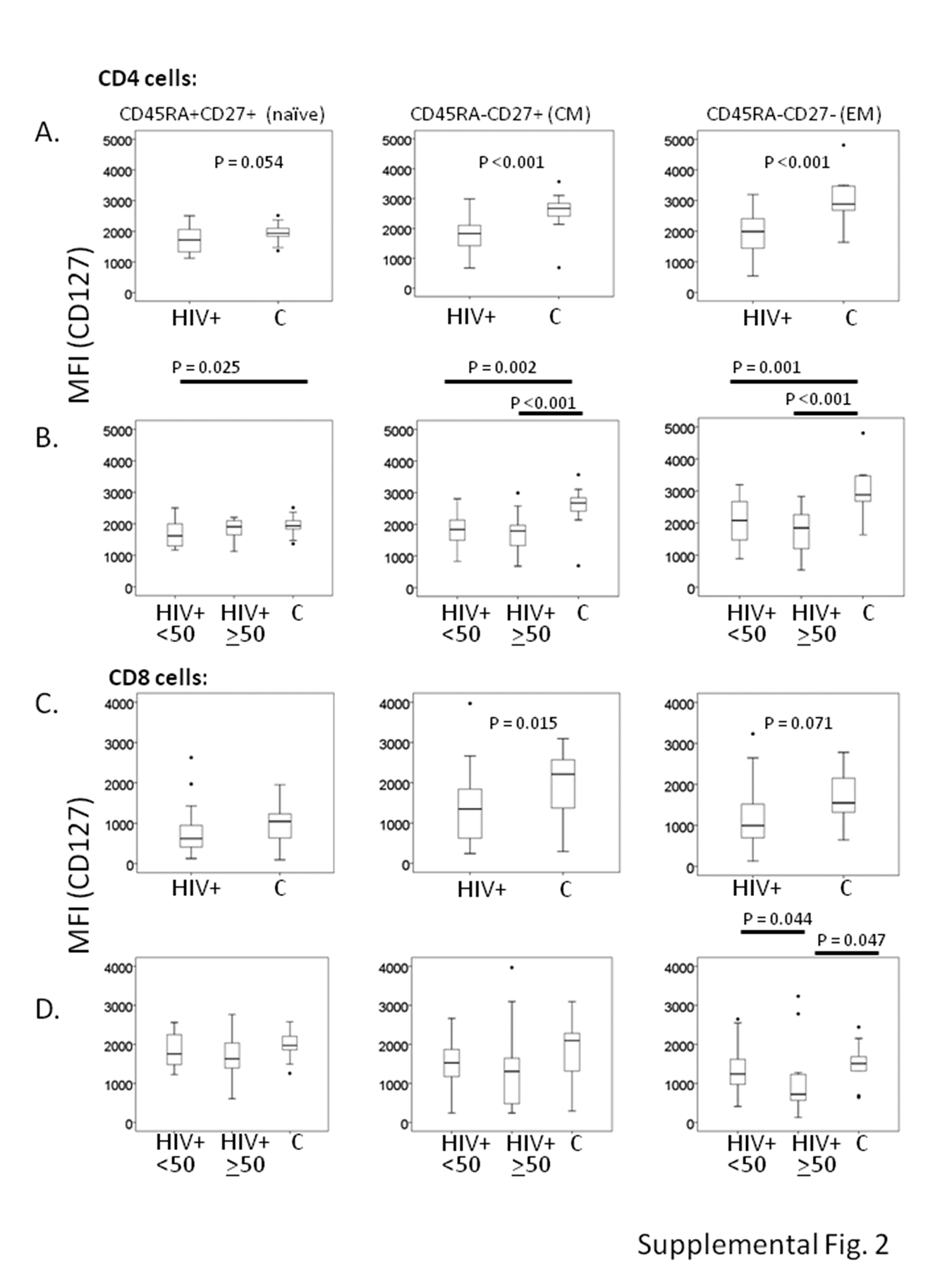

Supplement: Figure S2 — Reduced density of CD127 expression in T cells from HIV+ donors. Freshly isolated whole blood was stained for expression of CD127 in T cell subsets. The mean fluorescence intensity was measured specifically on CD127+ cells that were identified with an isotype control background stain. Box-and-whiskers plots are shown for MFI of CD127 expression in CD4+ T cells (A and B) and CD8+ T cells (C and D). Data represent all HIV+ donors (A and C) or virmeic and aviremic donors (B and D). (TIF) [file pone.0058764.s002.tif]

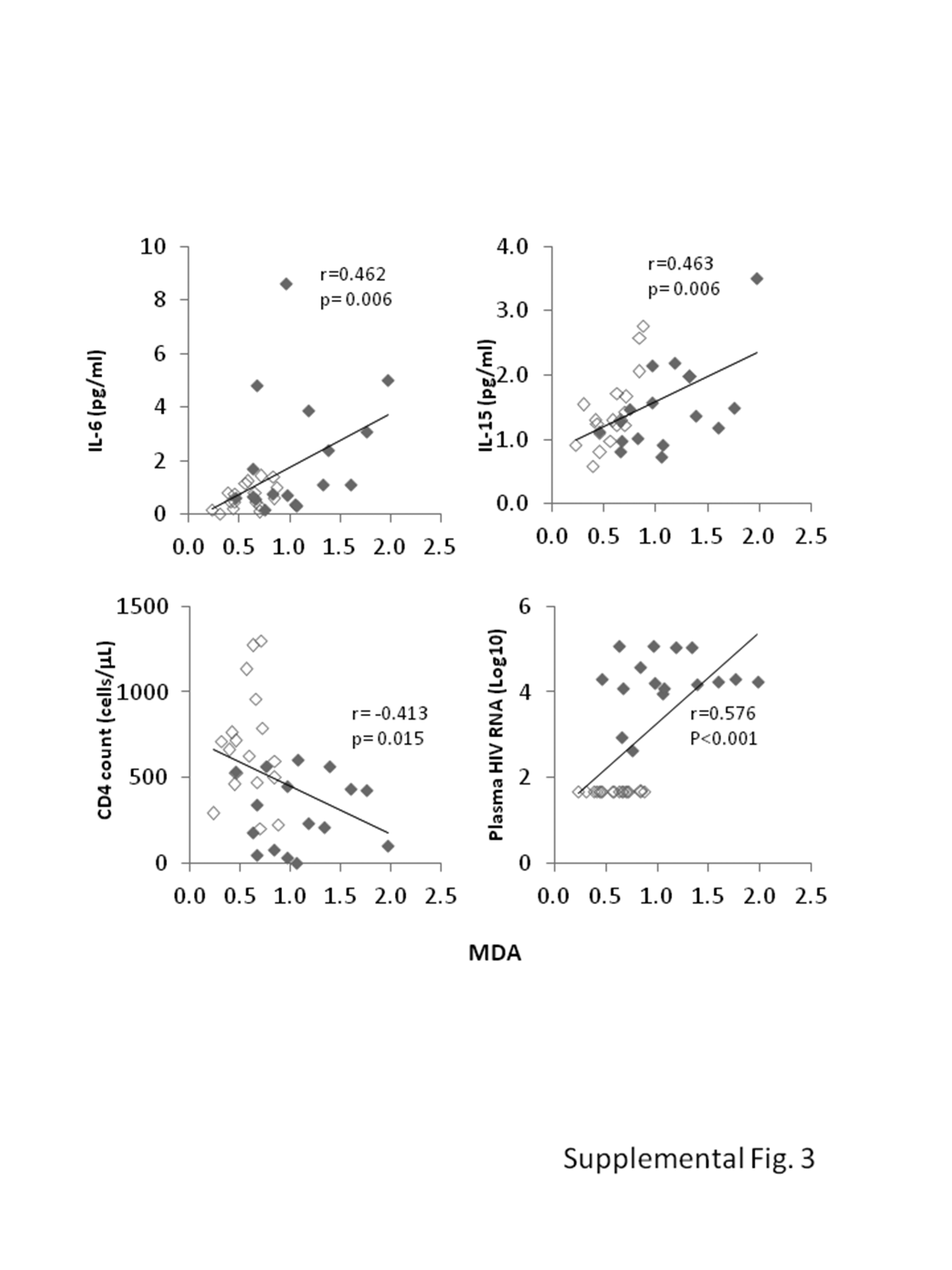

Supplement: Figure S3 — Serum MDA adducts are related to IL-6 and IL-15 cytokines and also correlated with clinical indices of disease progression. Serum concentrations of IL-6 and IL-15, CD4 T cell counts and plasma HIV RNA were plotted against serum MDA adducts in HIV+ donors. Open symbols represent aviremic subjects and closed symbols represent viremic subjects. Correlation coefficients and P values were determined by Spearman's correlations. (TIF) [file pone.0058764.s003.tif]

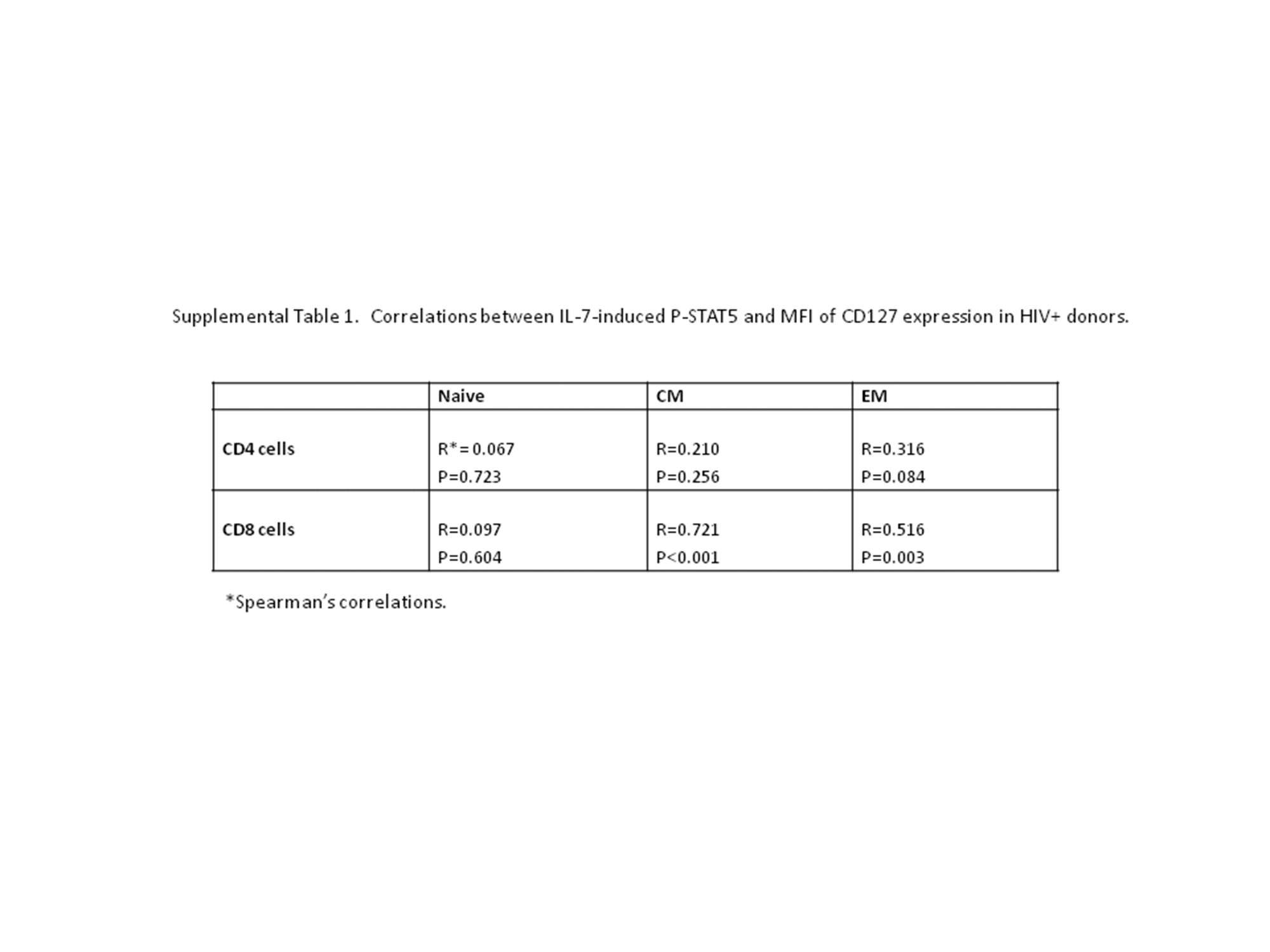

Supplement: Table S1 — Spearman's correlations indicating the relationships between CD127 MFI and P-STAT5 induction by IL-7 in T cell subsets from HIV+ donors. (TIF) [file pone.0058764.s004.tif]
